# Graphene–oxide interface for optoelectronic synapse application

**DOI:** 10.1038/s41598-022-09873-8

**Published:** 2022-04-07

**Authors:** Ricardo Martinez-Martinez, Molla Manjurul Islam, Adithi Krishnaprasad, Tania Roy

**Affiliations:** 1 NanoScience Technology Center, University of Central Florida, Orlando, FL 32826 USA; 2 Department of Electrical and Computer Engineering, University of Central Florida, Orlando, FL 32816 USA; 3 Department of Physics, University of Central Florida, Orlando, FL 32816 USA; 4 Department of Materials Science and Engineering, University of Central Florida, Orlando, FL 32816 USA

**Keywords:** Electrical and electronic engineering, Electronic devices, Two-dimensional materials

## Abstract

Optoelectronic synapses combine the functionalities of a non-volatile memory and photodetection in the same device, paving the path for the realization of artificial retina systems which can capture, pre-process, and identify images on the same platform. Graphene/Ta_2_O_5_/graphene phototransistor exhibits synapse characteristics when visible electromagnetic radiation of wavelength 405 nm illuminates the device. The photocurrent is retained after light withdrawal when positive gate voltage is applied to the device. The device exhibits distinct conductance states, modulated by different parameters of incident light, such as pulse width and number of pulses. The conductance state can be retained for 10^4^ s, indicating long term potentiation (LTP), similar to biological synapses. By using optical and electrical pulses, the device shows optical potentiation and electrical LTD repeatably, implying their applicability in neural networks for pattern recognition.

## Introduction

The miniaturization of digital components is reaching the limits predicted by Moore’s law, and new methodologies and devices that can collect, store and process information have been pursued. Motivated by the brain’s ability to store, process, and memorize information, neuromorphic computing rises as an active candidate to solve the miniaturization problem. Neuromorphic computing contrasts with traditional von Neumann architecture due to the well-suited interaction with sensory data in humanlike ways^1–4^. In biological brains, neuron and synapses are the smallest units of learning and memory. To do neuromorphic computing, the realization of neurons and synapses is necessary. Memristors have reached an advance stage of exploration for mimicry electronic synapses^5–8^. However, since most of the information sensed and stored by the brain comes from the eyes, a pure electrical synapse is not sufficient; an optoelectronic synapse is going to fulfill more necessities in the internet of things (IoT) and big-data era. For example, Chen et al.^9,10^, have shown an avalanche photodetector in the mid-wavelength infrared region (MWIR), which is important for remote sensing and defense^11^ because of its transparency in the Earth’s atmosphere; employing this type of photodetectors on optoelectronic synapses can improve the way for future pattern recognition applications. For the realization of an artificial retina, platforms integrating diffractive optics are required but chromatic aberration is a problem when using diffractive optics in such small scales. The work by Ou et al.^12^, shows an achromatic metasurface in the MWIR wavelengths compatible with CMOS platform^13^. By proper material engineering, the integration of this metasurfaces can help with the realization of artificial retina systems. Such multifunctional devices can perform photodetection and store the information carried by light signals, such as wavelength and intensity, as conductance states.

Realization of optoelectronic synaptic devices have been possible using 2D materials such as ${\mathrm{MoS}}_{2}$, ${\mathrm{WS}}_{2}$ and ${\mathrm{WSe}}_{2}$^14–18^. Graphene is another 2D material with unique optoelectronic properties that has not been fully explored for optoelectronic synapse applications. Graphene is a gapless two-dimensional (2D) material which exhibits an absorption spectrum from ultra-violet to terahertz wavelengths; it shows very large mobility and ambipolar characteristics^19–23^. Single-layer graphene absorbs only ~ 2.3% in visible and infrared regions and because of the zero band-gap behavior on the energy diagram, it leads to short lifetime of exciton in pure graphene; the high carrier mobility (electrons and holes) comes with the price of low light absorption, which gives low responsivity values ($1 \; \mathrm{mA}/\mathrm{W}$)^24,25^. Due to the semimetallic nature of graphene, a standard graphene field effect transistors with a graphene channel between two electrodes, can result in high dark current approaching the microampere regime which is an obstruction on many applications because of the increase in shot noise^26,27^. Hybrid graphene-quantum dot structures^26,28,29^, artificial nanostructures on graphene^30^ and functionalization of graphene^31^ have been used to increase the carrier lifetime in graphene-based photodetectors. Unfortunately, the use of quantum dots or nanostructures constrains the inherent wide absorption of graphene to that specific range of the assisted structure. Liu et al*.* used a dielectric layer between two monolayer graphene films to enhance the photocurrent by photogating effect caused by tunneling of photogenerated carriers from one layer to the other^25^. Qin et al., used a hybrid phototransistor based on monolayer graphene on top of single-walled carbon nanotubes (SWNTs) on a ${\mathrm{SiO}}_{2}/\mathrm{Si}$ substrate for optoelectronic synaptic device, the physical mechanism behind the operation being the interface trap between graphene and SWNTs^32^. Graphene-based optoelectronic synapse is also obtained by using 2D perovskite/graphene structure^33^. However, there has not been much exploration on the use of graphene/oxide interface for optoelectronic synapse which is important because it can get integrated easily with CMOS technology.

In this work, we use a graphene/Ta_2_O_5_/graphene heterostructure to demonstrate optoelectronic synapses*.* Ta_2_O_5_ has shown good platform on CMOS technology^34,35^. It also has a high energy bandgap of ~ 4 eV which facilitates the realization of optoelectronic synaptic characteristics in the visible spectrum^36^. It has been shown that Ta_2_O_5_ deposited by e-beam instead of RF sputtering gives a narrow gap of visible light absorption^34,35,37^. which is the mechanism used for deposition in this work. Graphene can show different mechanism for the change in current with light; photo-induced bolometric effect and photovoltaic^22^. In order to decouple these two effects, we used a Si/SiO_2_ substrate with 285 nm thick dielectric as opposed to Freitag et al.^19^ where the thickness of the dielectric is 90 nm. Probing the top graphene layer, we observe gate-controlled long-term potentiation (LTP) in these devices. A positive gate voltage is essential to enhance carrier lifetime, elongating the retention of photoconductance. The device exhibits distinct conductance states as a function of light pulse width and the number of light pulses applied. The device demonstrates LTP for $\ge$ 10^4^ s. It can be optically potentiated and electrically depressed to obtain its conductance weight update curves, essential for use in neural networks for pattern recognition.

## Results and discussion

**Figure 1:**
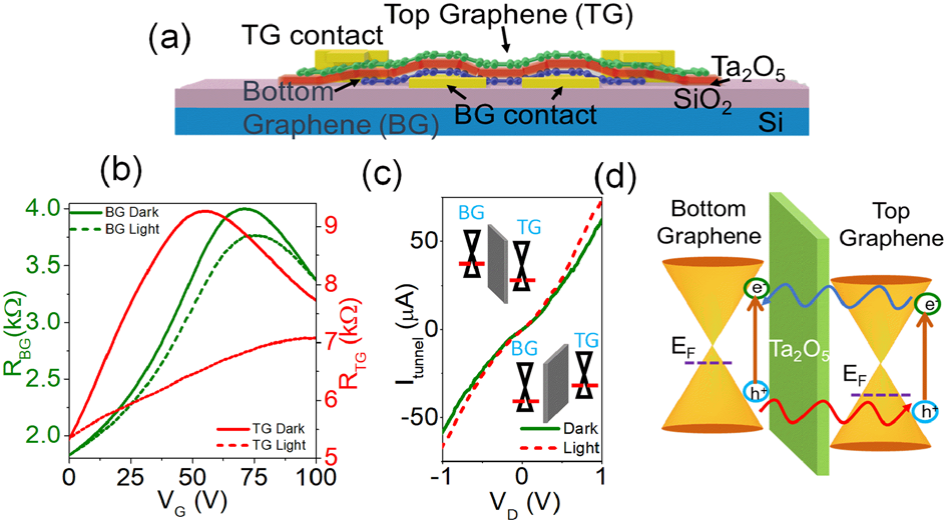
Graphene/Ta_2_O_5_/graphene (**a**) device schematic (not to scale). (**b**) *R vs. V*_*G*_ for bottom (*R*_*BG*_) and top (*R*_*TG*_) graphene in dark and under illumination of 405 nm wavelength. (**c**) Tunneling current from top graphene to bottom graphene layer as a function of drain source voltage ${V}_{D}$ between the two layers, while the substrate experiences a global back-gate voltage of 80 V, in dark and under illumination. The dielectric strength of SiO_2_ is 10^7^ V/cm and for Ta_2_O_5_ is $8\times {10}^{6}$ V/cm^43^. (**d**) Band diagram of graphene/Ta_2_O_5_/graphene heterostructure highlighting the mechanism of photocurrent generation on top graphene layer.

The graphene/Ta_2_O_5_/graphene device schematic is shown in Fig. 1a. The fabrication process is described thoroughly on the experimental section. Figure 1b shows the *R vs. V*_*G*_ characteristics of the bottom and top graphene films with the gate voltage applied through the Si substrate which we are going to refer as the global back gate voltage. Refer to methods for the thickness and doping of substrate. The hole mobility of the bottom graphene film in dark is calculated to be in the range of 1000–2272 cm^2^/V-s and the contact resistance in the range of 281–717 Ω for 10 devices. Figure S3 shows the transfer characteristics of 6 different devices. In order to calculate the mobility, we fit the $R \; \mathrm{vs} \; {V}_{G}$ plot using the model^27,38^:

$$R=2{R}_{c}+\frac{L}{Wq\mu }\frac{1}{\sqrt{{n}_{0}^{2}+{n}_{g}^{2}}}$$

where n_0_ ~ ε_r_εV_g_/te and at the charge neutrality point $R\approx 2{R}_{c}$^21,39^.

The *V*_*CNP*_ of the top graphene layer is lower than that of the bottom graphene layer. This indicates that the bottom graphene film is more p-doped than the top graphene film, given that the effective electric field reaching the bottom graphene is higher than the top graphene because of the additional Ta_2_O_5_ layer between the two graphene layers. Upon illumination with light of wavelength 405 nm, with a power of 100 mW, the *V*_*CNP*_ of both the top and bottom graphene layers shift positively. Figure 1c shows the current flowing from the top graphene layer to the bottom graphene layer (*I*_*tunnel*_) as a function of voltage applied across the two graphene layers, both in dark and under illumination, indicating the tunneling of carriers from one layer to the other across the Ta_2_O_5_ barrier. The tunneling current is enhanced upon illumination. During illumination, the bottom graphene layer generates electron–hole pairs. Since the bottom graphene layer has a higher charge neutrality point *V*_*CNP*_ than the top graphene layer in dark, implying a higher hole doping in the bottom graphene layer, the photogenerated holes tunnel into the top graphene layer. The photogenerated electrons from the top graphene layer can also tunnel into the bottom graphene layer. The accumulation of electrons in the bottom graphene layer leads to a photogating effect, shifting the *V*_*CNP*_ of the top graphene layer to more positive voltages. This enhances the photocurrent in the top graphene layer significantly, as indicated by the decreased resistance of the top graphene layer upon illumination. Because of the large spot area of the laser light it could also mean that near the graphene/metal interface causes a bolometric effect which in turn increases the photo-generated current. The mechanism of enhanced carrier generation due to the presence of a tunneling barrier between the top and bottom graphene layers is illustrated through the band diagram in Fig. 1d. The positive shift in *V*_*CNP*_ of both bottom and top graphene layers under illumination can also be associated to the adsorption of water molecules in the Gr/Ta_2_O_5_ interface. This behavior is not observed in Liu et al*.*, since RF sputtering creates a zero absorption Ta_2_O_5_ film^37^.

**Figure 2:**
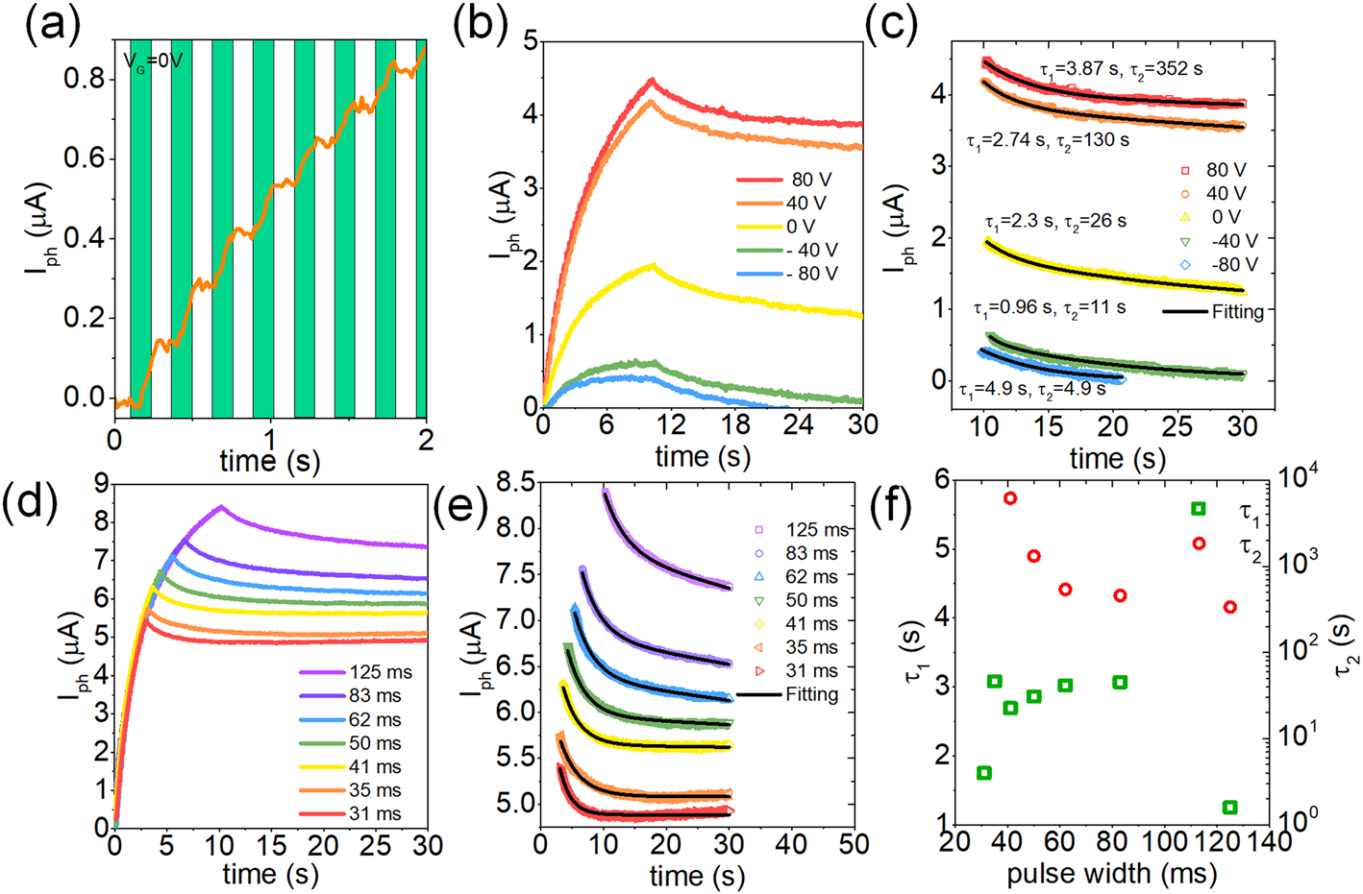
(**a**) Photocurrent measured across the top graphene layer as a function of time, when pulses of light (λ = 405 nm), of duration *t*_*ON*_ = 125 ms, *t*_*OFF*_ = 125 ms, are incident on the device. (**b**) Photocurrent measured across top graphene layer as a function of time, with varying back gate voltage. (**c**) Photocurrent retained after withdrawal of light as a function of time, fitted with a double exponential, indicating a fast decay with time constant τ_1_ followed by a slow decay of current with a time constant of τ_2_. (**d**) Photocurrent as a function of time with varying light pulse width. *V*_*G*_ = 80 V, *V*_*TGr*_ = 0.1 V. (**e**) Retained photocurrent after light pulse withdrawal as a function of time from (**d**), fitted with a double exponential. (**f**) Photocurrent decay time constants τ_1_ and τ_2_, extracted from (**e**), as a function of pulse width.

Figure 2a shows the effect of a stream of light pulses of 405 nm wavelength and intensity of $63 \; \mathrm{mW}/{\mathrm{cm}}^{2}$ incident on a typical graphene/Ta_2_O_5_/graphene device. The light pulses are 125 ms long. The back gate voltage *V*_*G*_ is held at 0 V. The bottom graphene is kept floating. A drain-source voltage of *V*_*TGr*_ = 0.1 V is applied to the top graphene layer. The dimensions of the bottom graphene layer is $L=10$ μm, $W=5$ μm and the dimensions of the top graphene layer is $L=44$ μm, $W=15$ μm. The calculated responsivity from Fig. 1b is: for the bottom graphene ${\mathcal{R}}_{b}=83.94 \; \mathrm{mV}/\mathrm{W}$, the calculated responsivity of top graphene is ${\mathcal{R}}_{t}=1.17 \; \mathrm{V}/\mathrm{W}$.

The photocurrent is given by *I*_*ph*_ = *I*_*light*_* – I*_*dark*_, where *I*_*dark*_ is the current through the top graphene layer in dark, and *I*_*light*_ is the current after illumination with each light pulse (shown in Fig. S4a). We observe that *I*_*ph*_ increases with each light pulse, and during the off period of 125 ms between two consecutive light pulses, the photocurrent does not decay to the dark state, indicating short term potentiation (STP) of the device. Figure 2b shows the effect of global-gate voltage on the LTP of the device. The global-gate voltage is varied from − 80 to + 80 V. The gate voltage is varied from − 80 to + 80 V. 40 light pulses, each with a duration of 125 ms, are applied on the device. The photocurrent, measured on the top graphene layer, increases with each incident pulse for all gate voltages. When the light pulses are withdrawn, the conductance state decays for *V*_*G*_ $\le$ 0 V, indicating STP. The total current under illumination is shown in Fig. S4b. The decaying photocurrent can be fitted by a double exponential function $Y={y}_{0}+{A}_{1}\mathrm{exp}(-t/{\tau }_{1})+{A}_{2}\mathrm{exp}(-t/{\tau }_{2})$ as shown in Fig. 2c. Here *y*_*0*_ is the offset, τ_1_ and τ_2_ are time constants for a fast and slow decay, respectively, and *A*_*1*_ and *A*_*2*_ are pre-exponentials. We observe that for all gate voltages, there is a fast (within ~ 3 s) decay in photocurrent. However, for positive gate voltages, there is a slow decay of conductance following that. The device is able to retain the conductance state for > 100 s. The photocurrent increases with increasing gate voltage, and the conductance retention increases as well. This is because, the positive back gate voltage helps in the trapping of electrons at the graphene/dielectric interfaces, leading to accumulation of holes in the top graphene layer. Figure 2d shows the effect of the light pulse width on the photocurrent and its retention. Figure S3c shows the total current under illumination. The pulse width is varied from 31 to 125 ms. The photocurrent increases with increasing pulse width, as expected, and decays only gradually after the pulses are withdrawn. Again, the decaying photocurrent is fitted with the aforementioned double exponential function as shown in Fig. 2e. The photocurrent undergoes a fast decay initially within the first ~ 3 s of light pulse withdrawal, and then relaxes into a steady level which retains for > 100 s, with τ_2_ reaching infinity for the pulse widths of 31 and 35 ms. The fitted time constants are plotted in Fig. 2f. This steady current level indicates the long-term potentiated conductance state of the device. These results illustrate that with a positive gate voltage, the Graphene/Ta_2_O_5_/Graphene devices can exhibit optically stimulated long term memory.

**Figure 3:**
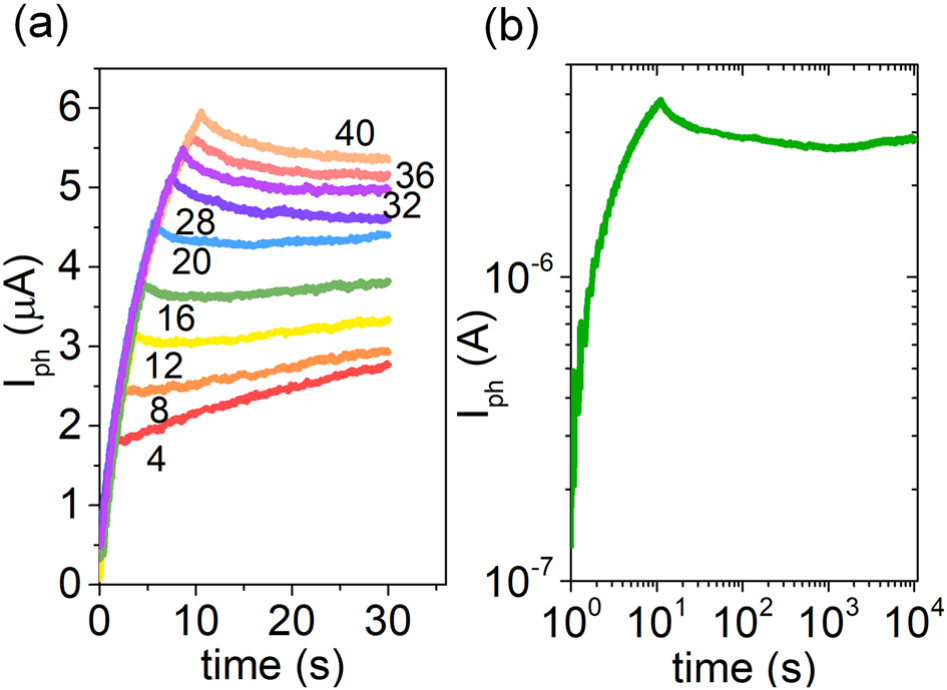
(**a**) Photocurrent across the top graphene layer as function of time for varying number of incident light pulses, wavelength = 405 nm, power = 100 mW, *t*_*ON*_ = *t*_*OFF*_ = 125 ms. (**b**) Long term potentiation for 10^4^ s, with 40 light pulses. *V*_*G*_ = 80 V, *V*_*TGr*_ = 0.1 V.

An electronic synapse shows multiple conductance states when subjected to electrical pulses. Similarly, a device behaves as an optoelectronic synapse when multiple non-volatile conductance states can be extracted as a function of number of incident optical pulses. Figure 3a shows the existence of 9 distinct conductance states for at least 20 s, in a typical Graphene/Ta_2_O_5_/Graphene device, as the number of light pulses is varied from 4 to 40. The gate voltage is held at + 80 V, and the photocurrent is measured from the top graphene layer held at a drain-source voltage (*V*_*TGr*_) of 0.1 V. Figure S4a shows the total current measured. Figure 3b shows that the device is able to retain its conductance state for ~ 10^4^ s after the application of 40 light pulses, each of duration 125 ms. The measured current through top graphene layer is shown in Fig. S4b. Typical electronic synapses exhibit a retention of > 10^3^ s for use in neural networks. Thus, our graphene/Ta_2_O_5_/graphene device is fit for practical implementation in neural networks for learning and inference tasks.

**Figure 4:**
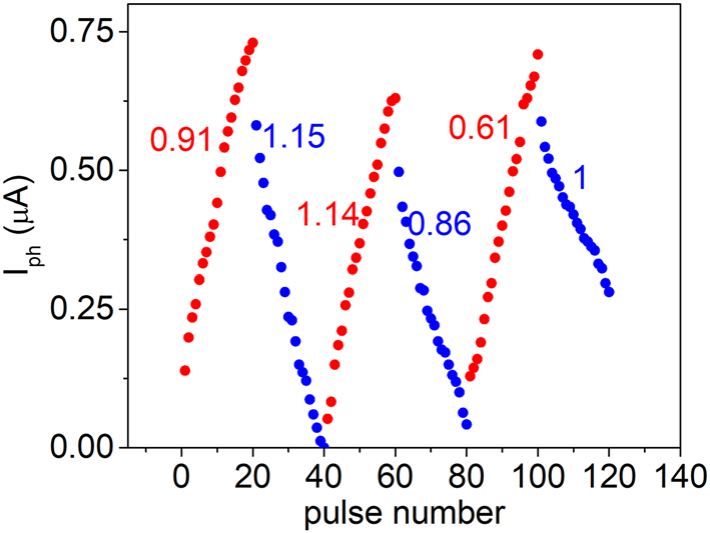
Weight update characteristics. Optical potentiation is obtained with 20 light pulses of t_ON_ = t_OFF_ = 125 ms, λ = 405 nm, *V*_*G*_ = 80 V, *V*_*TGr*_ = 0.1 V. Electrical depression is obtained in dark, keeping *V*_*TGr*_ at 0.1 V, and pulsing the gate voltage from 80 to 70 V, with a pulse width of 125 ms, using 20 voltage pulses. The cycle is repeated 3 times. Non-linearity factors for potentiation and depression are included in the plots.

A synaptic device’s conductance tuning curve is of utmost importance for its use in the training of a neural network. Linearity and symmetry in conductance weight update determine the accuracy of the neural network. For obtaining the weight update of the graphene/Ta_2_O_5_/graphene optoelectronic synapses, we use 20 optical pulses of duration 125 ms to potentiate the device, and 20 electrical pulses at the gate electrode to depress the device. While optically potentiating the device, the gate voltage *V*_*G*_ is held at + 80 V for inducing a condition of maximum conductance retention. To depress the device, the light pulses are withdrawn, and *V*_*G*_ is pulsed from + 80 to + 70 V, with a 125 ms on time and 125 ms off time. The top graphene drain-source voltage *V*_*TGr*_ is held at 0.1 V throughout the measurement. The reduction of the gate voltage using pulses releases trapped electrons in gradual steps and reduces the conductance of the device gradually. Thus, the conductance weight update curve is obtained, as shown in Fig. 4. The optical potentiation—electrical depression cycle is repeated 3 times to show the reliability of the process. We extract the nonlinearity factor (NLF) and symmetry of the potentiation and depression curves using a behavioral model described by Chen et al. The NLF values are close to 1, indicating a high linearity in weight update. Additionally, the symmetry, given by $\left|{NLF}_{potentiation}-{NLF}_{depression}\right|$ ranges from 0.2 to 0.4, indicating an extremely symmetric weight update, which further aids in improving the accuracy of the network.

## Conclusion

In summary, we report Graphene/Ta_2_O_5_/Graphene heterostructures acting as optoelectronic synapses. These devices show enhanced photocurrent due to photogating effect caused by selective tunneling of photogenerated carriers from one graphene layer to the other. The devices exhibit LTP, modulated by the gate voltage. The conductance states can be modulated by the light pulse width and the number of light pulses applied. The device retains its conductance state for 10^4^ s. Optical potentiation and electrical depression can be repeatably used to generate conductance weight update curves for these devices, to be used for the training of neural networks for pattern recognition tasks.

## Methods

### Materials and device fabrication

We first pattern the bottom graphene electrodes on a p-Si/SiO_2_ (285 nm) substrate, which acts as the global back gate, by standard photolithography technique, and deposit Ni/Au (60/20 nm) by electron beam evaporation followed by lift-off. A monolayer of graphene film, grown by chemical vapor deposition (CVD) on Cu foil is wet-transferred onto this substrate^40^, followed by annealing in forming gas (10% H_2_, 90% N_2_) for 3 h at 400 °C. The bottom graphene layer is patterned and etched in O_2_ plasma to form graphene strips. Next, 5 nm of Ta_2_O_5_ is patterned and deposited by e-beam evaporation. A second monolayer of CVD-grown graphene is wet-transferred on the substrate, patterned and etched into strips aligned with the bottom graphene layer. Ni/Au electrodes are patterned and deposited as contacts to the top graphene layer. Finally, the devices are annealed again in forming gas for 3 h at 300 °C.

### Device characterization

The optical image of a representative device with 5 terminals is shown in Fig. S1. The atomic force microscope (AFM) height profile shown in Fig. S2a confirms the thickness of the Ta_2_O_5_ film to be 5 nm. Figure S2b,c shows the Raman spectrum of the bottom and top graphene layers confirming that they are of monolayer thickness^41,42^. All electrical measurements were performed at room temperature on a probe station, using HP 4145B Semiconductor Parameter Analyzer and B1500A Semiconductor Device Analyzer. To measure the transfer characteristics after illumination of light, we use a cw laser diode light source in the visible spectrum radiation (${\lambda }_{0}=405 \; \mathrm{nm}$) where light propagates in free space (spot size 0.55 cm). For light pulses, the laser light travels through an optical fiber (77,563 newport fused silica) with a transmittance of 45% (spot area $\approx 0.714 \; {\mathrm{cm}}^{2}$).

## Supplementary Information

Supplementary Figures.

## Data Availability

All data generated and analyzed during this study are either included in the published article itself (or available within the Supplementary Information files).
