# Supplementary material for: Graphene–oxide interface for optoelectronic synapse application
Source: Sci Rep. 2022 Apr 7;12:5880. doi: 10.1038/s41598-022-09873-8 (PMC8991232; doi:10.1038/s41598-022-09873-8)
Supplement: Supplementary file 1 — Supplementary Figures. [file 41598_2022_9873_MOESM1_ESM.pdf]

Graphene-oxide interface for optoelectronic synapse application

Ricardo Martinez-Martinez,[1,2] Molla Manjurul Islam,[1,3] Adithi Krishnaprasad,[1,2] and Tania Roy[1,2,3,4]*

[1]*NanoScience Technology Center, University of Central Florida, Orlando, 32826, USA*

[2]*Department of Electrical and Computer Engineering, University of Central Florida, Orlando, 32816, USA*

[3]*Department of Physics, University of Central Florida, Orlando, 32816, USA*

[4]*Department of Materials Science and Engineering, University of Central Florida, Orlando, 32816, USA*

[*]*Corresponding author: tania.roy@ucf.edu*

Supporting Information

S1. Optical image for Graphene/Ta_2O_5/Graphene device

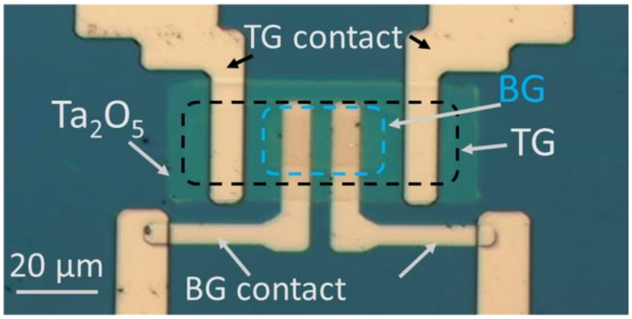

Figure S1. Optical image of a representative graphene/Ta_2O_5/graphene device.

Figure S1 displays the phototransistor based on graphene for optoelectronic synapse application. It shows four contacts made of Ni/Au (60/20 nm) and the back-gated contact. By selecting the biasing contacts, it is possible to vary the electronic properties of either graphene layer, i.e. bottom graphene (BG) or top graphene (TG). In here, the top graphene area is larger than the bottom one. The patterning of Ta_2O_5 is conducted by a standard photolithography process. The greenish color on the dielectric film Ta_2O_5 might be due to some photoresist remnants.

S2. Materials characterization on Graphene/Ta_2O_5/Graphene heterostructures

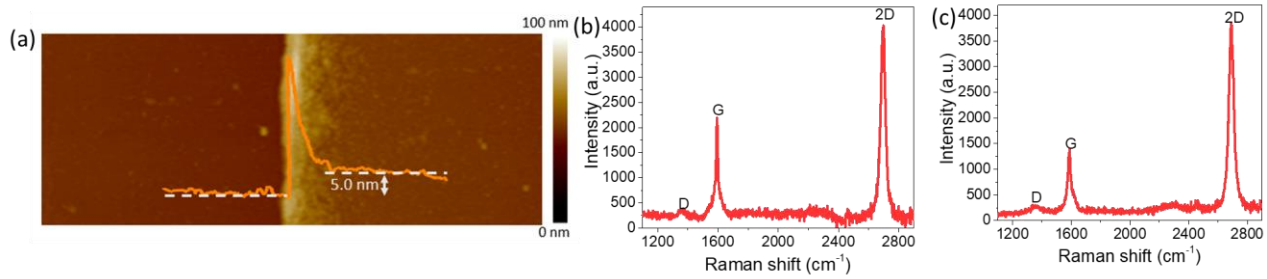

Figure S2. (a) AFM height profile of Ta_2O_5 in a graphene/Ta_2O_5/graphene heterostructure. Raman spectra of (b) bottom and (c) top graphene layers.

The height profile proves a homogenous film of 5nm of Ta_2O_5. It can be seen that the dielectric film of Ta_2O_5 isolates both graphene layers from each other and features the characteristics spikes of monolayer graphene for both layers.

S3. Transfer characteristics of BG and TG.

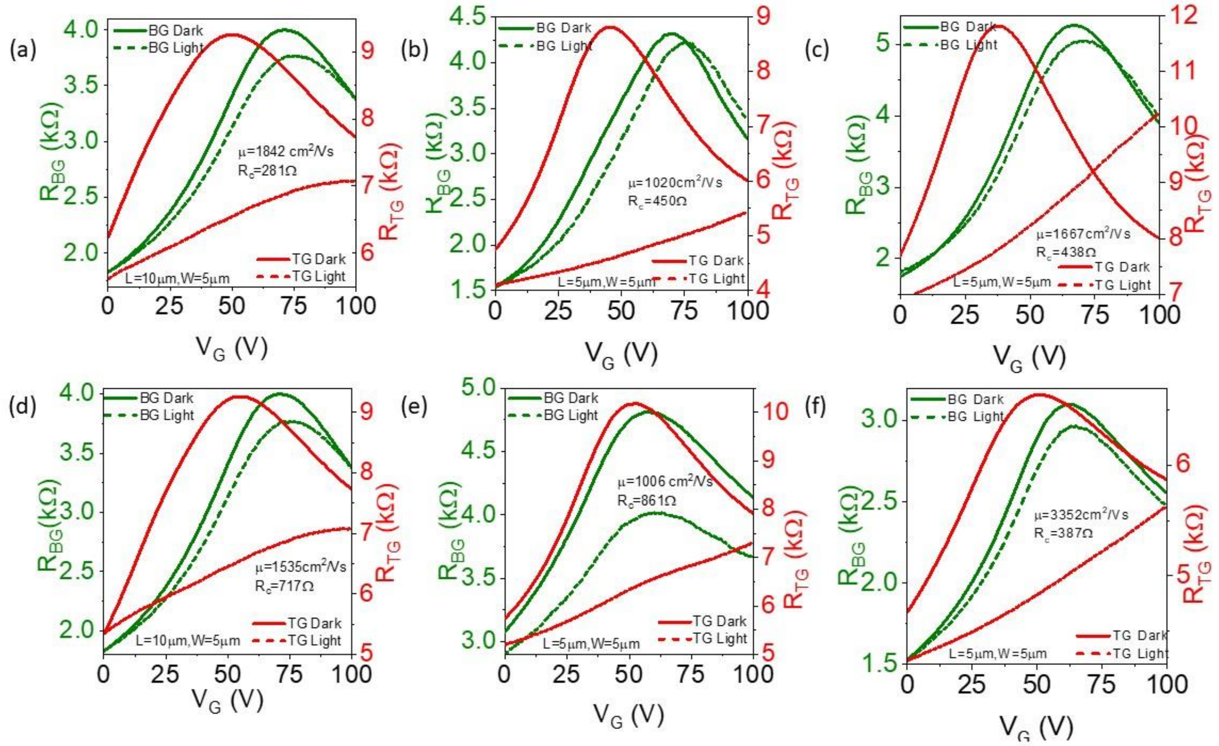

Figure S3. (a)-(f). $R - V_G$ plot of 6 different devices showing bottom graphene and top graphene layers in dark and light conditions.

Figure S3 shows the transfer characteristics of 6 different devices. For the bottom graphene, the charge neutrality point V_{CNP} appears on the same neighborhood. By fitting the bottom graphene resistance curve in dark, the mobility and contact resistance can also be calculated. The largest mobility computed is μ=3352 cm^2/Vs with a contact resistance of R_c=387 Ω.

S4 Gate tunability and light pulse width dependence on long term potentiation

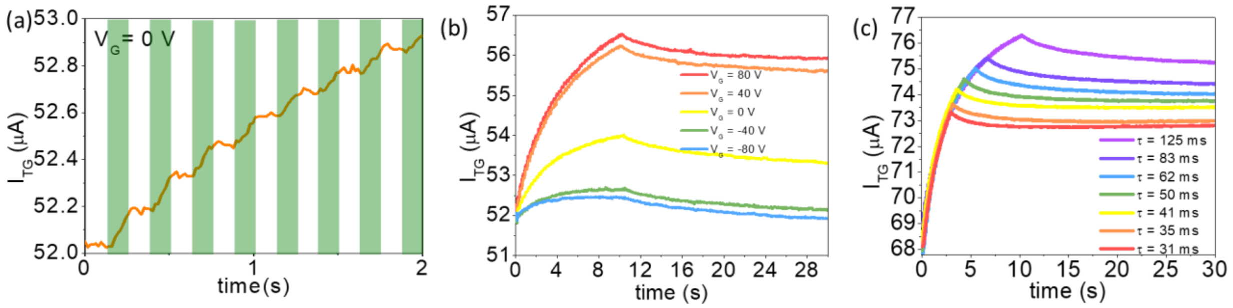

Figure S4. (a) Current measured across the top graphene layer as a function of time, when pulses of light (λ=405 nm), of duration t_{ON} = 125 ms, t_{OFF} = 125 ms, are incident on the device. (b) Current measured across top graphene layer as a function of time, with varying back gate voltage. (c) Current as a function of time with varying light pulse width. V_G = 80 V, V_{TGr} = 0.1 V.

In Figure S4 a, when illumination is off, the current does not go down immediately, rather it stays on the same level and increases with the following pulse. The PSC (post-synaptic current) stays after illumination is turned off as shown in Figure S3 b. The device also shows plasticity by varying the pulse width as seen in Figure S4 c.

S5. Effect of number of light pulses on LTP and LTP of 10^4 s

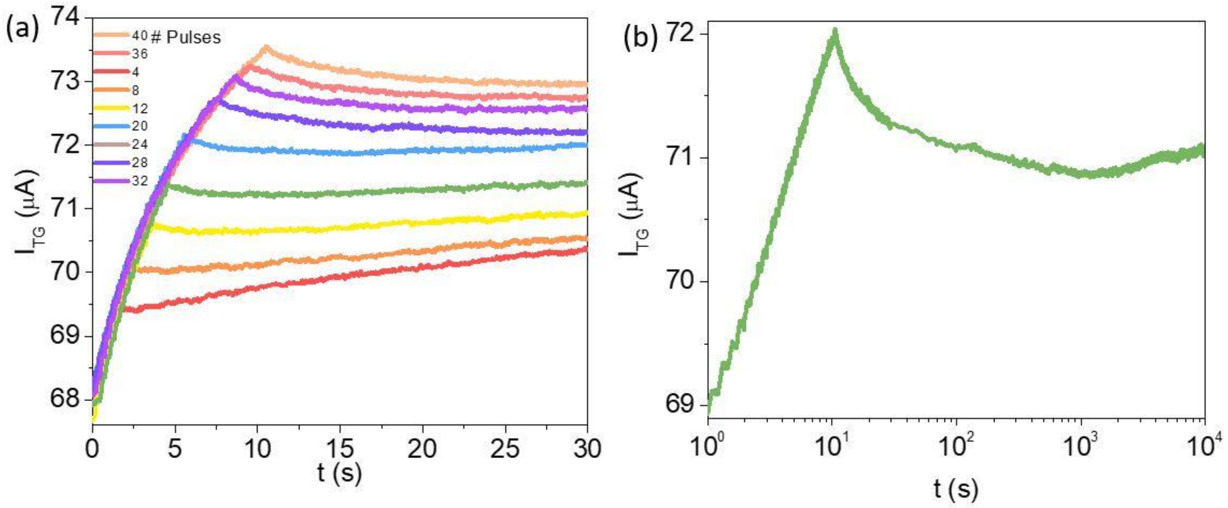

Figure S5. (a) Total current across the top graphene layer as a function of time for varying number of incident light pulses, wavelength = 405 nm, power = 100 mW, $t_{ON} = t_{OFF}$ = 125 ms.. (b) Long term potentiation for 10^4 s, with 40 light pulses. V_G = 80 V, V_{TGr} = 0.1 V.

The plasticity on this device is clearly observed when the number of pulses changes from 4 up to 40. The long-term potentiation is a feature of biological synapses and it is extremely important for pattern recognition in futures AI platforms.

S6. Optical potentiation and electrical depression

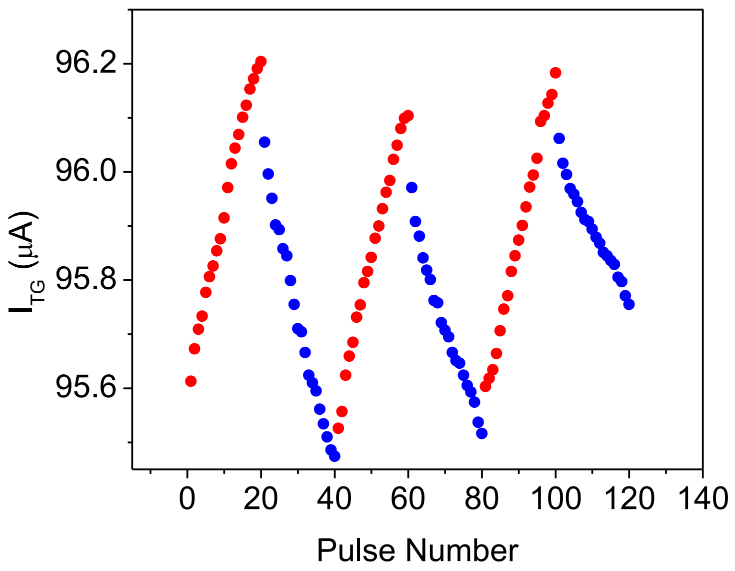

Figure S6. Weight update characteristics showing the total measured current.

In Figure S6, 20 optical pulses are applied to potentiate the device and 20 electrical pulses are applied to decrease the current level. The device shows 3 cycles of potentiation/depression.
